# Current and emerging medical and surgical therapy in hypertrophic cardiomyopathy

**DOI:** 10.1186/s44348-025-00050-9

**Published:** 2025-09-24

**Authors:** Kyung An Kim, Mi-Hyang Jung

**Affiliations:** 1 Division of Cardiology, Department of Internal Medicine, Incheon St. Mary’s Hospital, The Catholic University of Korea, Incheon, Republic of Korea; 2 Catholic Research Institute for Intractable Cardiovascular Disease, College of Medicine, The Catholic University of Korea, Seoul, Republic of Korea; 3 Division of Cardiology, Department of Internal Medicine, Seoul St. Mary’s Hospital, The Catholic University of Korea, Seoul, Republic of Korea

**Keywords:** Hypertrophic cardiomyopathy, β-blockers, Nondihydropyridine calcium channel blockers, Sodium-glucose cotransporter 2 inhibitors, Cardiac myosin inhibitors, Septal reduction therapy, Gene-targeted therapy

## Abstract

Hypertrophic cardiomyopathy (HCM) is a disease characterized by unexplained left ventricular hypertrophy and is caused by mutations in cardiac sarcomeric proteins. Despite advances in diagnostic modalities and risk stratification, therapeutic strategies have until recently mostly focused on the management of symptoms and the prevention of sudden cardiac death, rather than modifying the underlying sarcomeric dysfunction itself. Conventional pharmacological therapies such as β-blockers and nondihydropyridine calcium channel blockers are effective first-line treatments for obstructive HCM, and established invasive septal reduction therapies, such as surgical myectomy and alcohol septal ablation, provide effective relief of obstruction in refractory patients. However, these therapies address anatomical and hemodynamical consequences rather than the molecular etiology of the disease. In recent years, novel therapeutic approaches have emerged that target the pathophysiological mechanisms of HCM more directly. Sodium-glucose cotransporter 2 inhibitors have demonstrated clinical benefits in HCM through improvements in myocardial energetics. Cardiac myosin inhibitors directly attenuate sarcomeric hypercontractility and have shown improvements in symptoms, functional status, and hemodynamic parameters in obstructive HCM. Furthermore, preliminary gene-targeted therapies are under active investigation and offer the prospect of definitive cure. This review provides a comprehensive overview of current and emerging treatment modalities for HCM. Overall, the management of HCM is evolving toward a more mechanism-targeted approach spanning from gene to myocardium. Ongoing research will be essential to integrate the emerging molecularly targeted therapies with established management strategies into a personalized, multidisciplinary management of HCM.

## Background

Hypertrophic cardiomyopathy (HCM) is the most common genetic cardiomyopathy, traditionally estimated to affect about 1 in 500 people in the general population [1–3]. HCM is defined by left ventricular hypertrophy (wall thickness ≥ 15 mm in any myocardial segment or ≥ 13 mm in genetic carriers) which is not solely explained by abnormal loading conditions [4, 5]. The disease is often inherited in an autosomal dominant pattern, most frequently caused by pathogenic variants in sarcomeric protein genes (such as β-myosin heavy chain *MYH7* or myosin-binding protein C *MYBPC3*) [4, 6]​. Details on the genetics and molecular pathophysiology of HCM, as well as diagnosis using multimodality imaging, have been published previously in this review series; in this article, our focus will be on the pharmacologic and surgical management of HCM.

Currently, the treatment objectives in HCM are threefold: (1) alleviate symptoms (e.g., dyspnea, chest pain, fatigue) by reducing left ventricular outflow tract (LVOT) obstruction and improving diastolic filling; (2) prevent sudden cardiac death—largely through risk stratification for implantable cardiac defibrillators; and (3) control atrial fibrillation, which impairs quality of life and is a potential turning point for excess mortality [4, 7–9]. The prevention of sudden cardiac death and management of atrial fibrillation are discussed at length in other articles in this review series. Historically, management has focused on symptom control and sudden cardiac death prevention because no therapy could directly reverse the underlying hypertrophy or sarcomeric dysfunction [10]. Standard pharmacological treatments (e.g., β-blockers, nondihydropyridine calcium channel blockers [CCBs]) improve exercise tolerance but do not modify the disease substrate. Invasive septal reduction therapies (surgical myectomy or alcohol septal ablation [ASA]) can effectively relieve LVOT gradients in obstructive HCM, but these are targeted to anatomy rather than the genetic cause. Thus, despite advances, limitations of existing therapies include an inability to fundamentally reverse the root causes of HCM—namely, the sarcomere hypercontractility, energy dysregulation, and cellular signaling pathways driving hypertrophy and fibrosis [11].

In recent years, new treatment strategies have emerged to fill this gap. These include sodium-glucose cotransporter 2 (SGLT2) inhibitors that unexpectedly confer cardiac benefits [12], and precision medicine approaches targeting the molecular defects. The introduction of mavacamten—a first-in-class cardiac myosin inhibitor—represents a milestone, as it directly counteracts the hypercontractility of HCM​ [13–15]. Furthermore, experimental gene editing therapies offer the prospect of curing HCM by correcting or silencing mutant alleles, as shown in preclinical models​ [16–18]. Overall, the field of HCM treatment is on the brink of rapid development after decades of limited options [10]. In this review we will examine the current state of HCM management, including traditional treatments (both pharmacologic and invasive) and also explore novel therapies on the horizon.

### Pharmacological management

Conventional medical therapy in HCM has long centered on negative inotropic and chronotropic agents to mitigate LVOT obstruction and to improve diastolic filling for symptom relief. β-Blockers, nondihydropyridine CCBs, and disopyramide are the pharmacologic agents that have been traditionally used in obstructive HCM. By reducing heart rate and contractility, these drugs decrease LVOT obstruction and the systolic anterior motion of the mitral valve, thereby providing symptom relief [19, 20]. Guidelines recommend optimization of medical treatment in obstructive HCM patients before proceeding to more invasive options such as septal reduction therapy (SRT) [4, 5]. In contrast, treatment options for nonobstructive HCM have up until recently been more limited because of a lack of data to suggest that medical therapy alters the natural history of the disease [5]. In this section we first review the role of traditional pharmacotherapy in the treatment of HCM, and cover new pharmacologic agents including SGLT2-inhibitors and cardiac myosin inhibitors.

### Traditional pharmacotherapy (β-blockers, nondihydropyridine CCBs, disopyramide)

Nonvasodilating β-blockers (e.g., propranolol, metoprolol, bisoprolol) are first-line therapy for symptomatic obstructive HCM, with a class I recommendation in both American College of Cardiology (ACC)/American Heart Association (AHA) [5] and European Society of Cardiology (ESC) guidelines [4, 5] β-Blockade slows heart rate, prolongs diastole, and reduces contractility, which increases end-diastolic volume and reduces the degree of LVOT obstruction. Of note, β-blockers with more pronounced vasodilatory properties (e.g., carvedilol, nebivolol) may theoretically worsen LVOT obstruction due to decreases in afterload [21]. Decades of clinical use have demonstrated the benefits of β-blockers in improving exercise capacity and alleviating angina [19, 22–26]. However, as most of the studies demonstrating the efficacy of β-blockers were small, nonrandomized, and conducted a long time ago, there is relatively little high quality evidence to support β-blocker treatment [10].

In a recent, blinded, placebo-controlled crossover trial, metoprolol lowered LVOT gradients and improved New York Heart Association (NYHA) functional class as well as stroke volume and angina symptoms in patients with obstructive HCM [27, 28]. Of note, there was no improvement in measures of exercise capacity including peak oxygen consumption, which may have been due to the negative chronotropic effects of β-blockade [27]. In an analysis of data from the EXPLORER-HCM and MAVA-LTE studies, mavacamten improved LVOT gradients, exercise capacity, and symptom burden independent of β-blocker use, while β-blockers was often associated with chronotropic incompetence leading to a reduction in peak oxygen consumption [29]. This raises the hypothesis that mavacamten monotherapy may be a reasonable option to eliminate the various side effects from β-blocker treatment [27, 29], and whether β-blockers will retain its place as the first-line therapy for obstructive HCM in the future remains to be seen.

Nondihydropyridine CCBs such as verapamil and diltiazem are considered second-line or adjunctive agents if β-blockers are insufficient or not tolerated​, with a class I recommendation in both ACC/AHA and ESC guidelines [4, 5]. As with β-blockers, the beneficial effects of these medications are largely due to negative inotropic and chronotropic effects [10, 30]. Verapamil in particular has shown symptomatic benefit in HCM patients, increasing exercise tolerance and reducing the frequency of chest pain [31–34]. However, care must be taken in severe obstructive HCM, as the vasodilatory effect of CCBs can reduce afterload and potentially worsen the gradient [35]. Also, dihydropyridine CCBs such as amlodipine or nifedipine, as well as other vasodilators such as renin-angiotensin system blockers and nitrates, may exacerbate LVOT obstruction and are therefore relatively contraindicated​ in obstructive HCM [4, 5, 36, 37], although the safety of renin-angiotensin system blockers has been reported in several studies [38, 39].

Disopyramide, a class IA antiarrhythmic agent, can lower LVOT gradients in obstructive HCM [40, 41], as its sodium-channel blocking properties can reduce myocardial contractility significantly [42]. Randomized trial data on disopyramide are limited, but observational studies show improvement in LVOT gradients, symptoms, and exercise capacity in patients who are symptomatic despite treatment with β-blockers and nondihydropyridine CCBs [43–45]. Because of vagolytic side effects and the risk of arrhythmias, disopyramide is usually reserved as an add-on therapy for patients who are refractory to first-line therapy [45, 46]. Disopyramide has a class I recommendation in both ACC/AHA and ESC guidelines for obstructive HCM patients who have persistent symptoms despite β-blockers and nondihydropyridine CCBs [4, 5]. However, concerns over its significant side effects limit its use, and many HCM centers use disopyramide mainly as a short-term bridge to other treatments such as surgical myectomy [10]. Of note, disopyramide is currently unavailable in the Republic of Korea.

For nonobstructive HCM, β-blockers and nondihydropyridine CCBs may also help by slowing heart rate, improving the relation between filling time and relaxation, and improving diastolic function [47]. These agents have shown efficacy in reducing dyspnea and angina, and improving exercise capacity and myocardial perfusion [34, 48–52]. Both β-blockers and nondihydropyridine CCBs have a class I recommendation in both ACC/AHA and ESC guidelines [4, 5]. However, they have only been evaluated in a few small studies, and their use is mostly based on extrapolation from obstructive HCM [10]. When symptoms persist even after treatment with β-blockers and nondihydropyridine CCBs, diuretics may be cautiously tried to improve dyspnea and volume status [5]. In addition, SGLT2 inhibitors may also be appropriately considered based on heart failure indications, as will be discussed in the following section.

Overall, traditional pharmacotherapy is aimed at symptomatic relief. While often effective in improving quality of life, these drugs do not reduce hypertrophy or fibrosis and do not prevent disease progression in many cases. This has prompted investigation into novel pharmacological agents that could modify HCM at the myocardial level.

### SGLT2 inhibitors

SGLT2 inhibitors are oral antihyperglycemic agents originally developed for type 2 diabetes. However, beyond glucose control, SGLT2 inhibitors have improved outcomes in heart failure, including those without diabetes, due to mechanisms that extend beyond glycemic effects [53–56]. Given that HCM often features diastolic dysfunction and microvascular ischemia, researchers have hypothesized that SGLT2 inhibitors might confer benefit in HCM as well. In the context of HCM’s pathophysiology, two major factors are diastolic dysfunction and energetic inefficiency in the hypertrophied myocardium [57]. SGLT2 inhibitors may improve cardiac energetics by shifting myocardial metabolism from glucose towards ketone utilization, thereby enhancing adenosine triphosphate availability for better diastolic function [58, 59]. They also have been shown to reduce intracellular sodium and calcium overload in cardiomyocytes, which could improve relaxation and reduce arrhythmogenicity [60, 61]. In a recent study, SGLT2 inhibitors directly enhanced myocardial relaxation and contractile function in human engineered heart tissues with HCM mutations [57].

Clinical studies have also provided evidence supporting the beneficial effects of SGLT2 inhibitors in HCM. A prospective open-label study evaluating empagliflozin in patients with coexistent type 2 diabetes and nonobstructive HCM found that the empagliflozin group showed significant improvements in E/e’ and E/A ratios, as well as NYHA class improvement [62]. In a propensity-matched study of over 4,000 patients using the Korean National Health Insurance Service database, HCM patients receiving SGLT2 inhibitors had significantly lower rates of all-cause death and heart failure hospitalization compared to those on other antidiabetic regimens​ [12]. Over a median 3.1-year follow-up, SGLT2 inhibitor use was associated with a 24% relative risk reduction in the composite of death or heart failure admission, with notably lower all-cause mortality and risk of sudden cardiac death (hazard ratio of 0.56 and 0.50, respectively). Similar findings were found in another retrospective health record-based study [63]. The ongoing SONATA-HCM trial (ClinicalTrials.gov identifier: NCT06481891) is a phase 3 randomized study testing a dual SGLT1/2 inhibitor, sotagliflozin, in patients with symptomatic HCM. This trial aims to assess improvements in exercise capacity, symptoms, and patient-reported outcomes, and will provide more definitive evidence on whether SGLT2 inhibitors can be a disease-modifying strategy in HCM.

### Myosin inhibitors

Cardiac myosin inhibitors such as mavacamten and aficamten have emerged as promising agents for obstructive HCM. In the EXPLORER-HCM trial, mavacamten significantly reduced LVOT gradients and improved exercise capacity in symptomatic patients with obstructive HCM, and in the VALOR-HCM trial, mavacamten also reduced the proportion of patients meeting LVOT gradient criteria for SRT [13, 14]. The REDWOOD-HCM trial also showed that aficamten significantly reduced LVOT gradients and N-terminal pro-brain natriuretic peptide levels in obstructive HCM [64]. These medications act by limiting the formation of actin-myosin cross-bridge formation, which in turn decreases contractility and enhances myocardial energy efficiency [65]. Given their expanding evidence base, a more detailed discussion is provided in a separate review.

### Emerging therapies

Given HCM’s status as a genetic disease of the cardiac sarcomere, there has been interest in therapies that target the genotype directly. The goal of genetic therapy is to move beyond treating the downstream effects of HCM and instead correct or silence the mutant genes that cause the disease [66]. Recent advances in molecular biology—including gene transfer vectors, RNA-based therapies, and gene editing tools—have opened the door to precision treatments for inherited cardiomyopathies. Here, we review the cutting-edge developments in genetic therapy for HCM, focusing on adeno-associated virus (AAV)-based gene delivery and genome editing approaches, as well as how genotype–phenotype correlations inform these strategies. We also highlight ongoing clinical trials translating these innovations to patients.

### AAV-mediated gene replacement

One approach to HCM gene therapy is delivering a healthy copy of a gene to supplement or replace a defective one. This is especially relevant for HCM mutations that lead to haploinsufficiency (insufficient protein due to a truncation or null allele). Notably, the majority of MYBPC3 mutations result in unstable or absent cardiac myosin-binding protein C due to only one functional gene copy, ultimately causing hypertrophy [67, 68]. In preclinical models, using AAV vectors to deliver a functional MYBPC3 gene to the heart has shown promise. Mice with MYBPC3 gene knockout or mutation, when treated with AAV-MYBPC3 gene transfer, demonstrated improvement of cardiac function and long-term prevention of cardiac hypertrophy [69–71]. In cardiomyocyte models engineered from human embryonic stem cells carrying a MYBPC3 mutation, AAV-mediated MYBPC3 gene delivery brought cardiac myosin-binding protein C levels to wild-type levels, and were also able to prevent hypertrophy and sarcomere disarray [72, 73]. Building on such data, a biotech initiative led to the development of TN-201, an AAV-based gene therapy encoding human MYBPC3. This therapy is designed as a one-time infusion that transduces cardiomyocytes to produce normal myosin-binding protein C, thereby addressing the protein deficit in MYBPC3-mutation HCM. In late 2023, a landmark first-in-human trial (MyPeak-1) of TN-201 was launched in adults with MYBPC3-associated HCM (ClinicalTrials.gov identifier: NCT05836259). Preliminary results show that low-dose TN-201 was well-tolerated in the initial three patients, with no significant cardiac adverse events or arrhythmias attributable to the therapy.

### RNA therapies and allele silencing

Not all HCM mutations are best treated by adding a gene—some may be better addressed by silencing an abnormal gene product. For instance, certain missense mutations produce a protein that disrupts sarcomere function via a dominant-negative effect [6]. In such cases, turning off the mutant allele could theoretically prevent HCM. Antisense oligonucleotides (ASOs) and RNA interference are tools to selectively suppress specific messenger RNA transcripts, and have received much attention in the development of new drugs in other diseases such as dyslipidemia [74, 75]. In mouse models, allele-specific silencing of mutant MYH6 suppressed the development of HCM [76], and exon skipping therapy to skip a mutated exon in the MYBPC3 gene using ASOs reduced levels of incorrectly spliced messenger RNA, producing stable functional proteins and preventing cardiac hypertrophy [77]. RNA-based strategies are still in preclinical stages for HCM, but rapid advances in oligonucleotide therapeutics may soon make them viable for certain genetic subsets.

### CRISPR gene editing and base editing

Perhaps the most exciting and technically challenging approach is to edit the mutant DNA itself. CRISPR/Cas9 (clustered regularly interspaced short palindromic repeats–CRISPER-associated protein 9) gene editing has been employed in research models to correct HCM mutations at the genomic DNA level [66, 78, 79]. Two seminal preclinical studies (published back-to-back in *Nature Medicine* in 2023) used CRISPR-Cas9 gene editing to fix a classic MYH7 mutation (c.1208G > A; p.Arg403Gln). Chai et al. [16] utilized a CRISPR-Cas9 adenine base editor (ABE) delivered by a dual AAV system to convert the mutant nucleotide back to the normal sequence in vivo. AAV-ABE injection achieved up to 35% correction of the mutant allele at the transcript level with minimal bystander edits of the wild-type allele. To test the efficacy of gene therapy in phenotype rescue, the authors developed a humanized mouse model with the MYH7 c.1208G > A mutation, with the effect that heterozygous mice developed cardiac hypertrophy and fibrosis at 12 weeks, while homozygous mice exhibited severe cardiomyopathy at birth and usually died within 1 week. Gene editing was able to extend the lifespan of homozygous mice to 2 weeks, while in heterozygous mice, AAV-ABE injection was able to prevent the onset of cardiac hypertrophy up to 16 weeks after birth.

Reichart et al. [17] also used a dual AAV system to deliver ABE and guide RNA targeting the same mutation, but with a different promoter to maximize editing efficiency. They achieved up to 70% transcript correction of the pathogenic allele in mice cardiomyocytes, with corresponding improvements in cardiac hypertrophy and fibrosis. However, in contrast to Chai et al. [16], significant bystander edits were detected, possibly due to the use of a less specific ABE and promotor sequence maximizing efficiency [66]. Reichart et al. [17] also demonstrated the efficacy of a traditional CRISPR nuclease approach using *Staphylococcus aureus* Cas9 to selectively knock out the mutant gene copy. This approach successfully disrupted approximately 40% to 60% of the mutant alleles, but at high vector doses some off-target cutting of the wild-type allele occurred, leading to reduced contractility in those cases.

These studies illustrate both the potential and challenges of gene editing for HCM: in principle, a one-time injection of gene editors early in life could “cure” HCM by fixing the mutation causing phenotype expression, as evidenced by improved cardiac function and disease-free survival in treated mice. On the other hand, delivery efficiency and specificity, as well as the adverse effects from off-target edits, functional mosaicism from incomplete editing, and immune reactions to AAV-ABE are major hurdles before this can be applied to human patients [18, 66].

### Future outlook

As these gene-targeted therapies develop, one obvious consideration is the specific genotype of the HCM patient. HCM is genetically heterogeneous—over 30 genes have been implicated and even within a single gene, different mutations may cause different clinical courses [6, 80]. Even now, guidelines recommend genetic testing for all HCM patients, partly to screen family members, but increasingly this information will have therapeutic relevance [4]. In the future, genotype–phenotype correlations may inform not only risk stratification but also which therapy the patient is likely to benefit from [81, 82]. Genetic therapies hold the promise of precision medicine for HCM, potentially transforming management from lifelong symptom control to one-time cures or disease-preventing interventions [83]. As these therapies progress, the landscape of HCM treatment may shift from predominantly pharmacological and interventional management to a genotype-driven approach. However, gene therapy in HCM is still in the early stages of development, with challenges such as effective delivery, off-target effects, and immune response modulation still remaining to be overcome, as well as ethical concerns over genetic manipulation [18].

### Invasive and surgical interventions

**Fig. 1:**
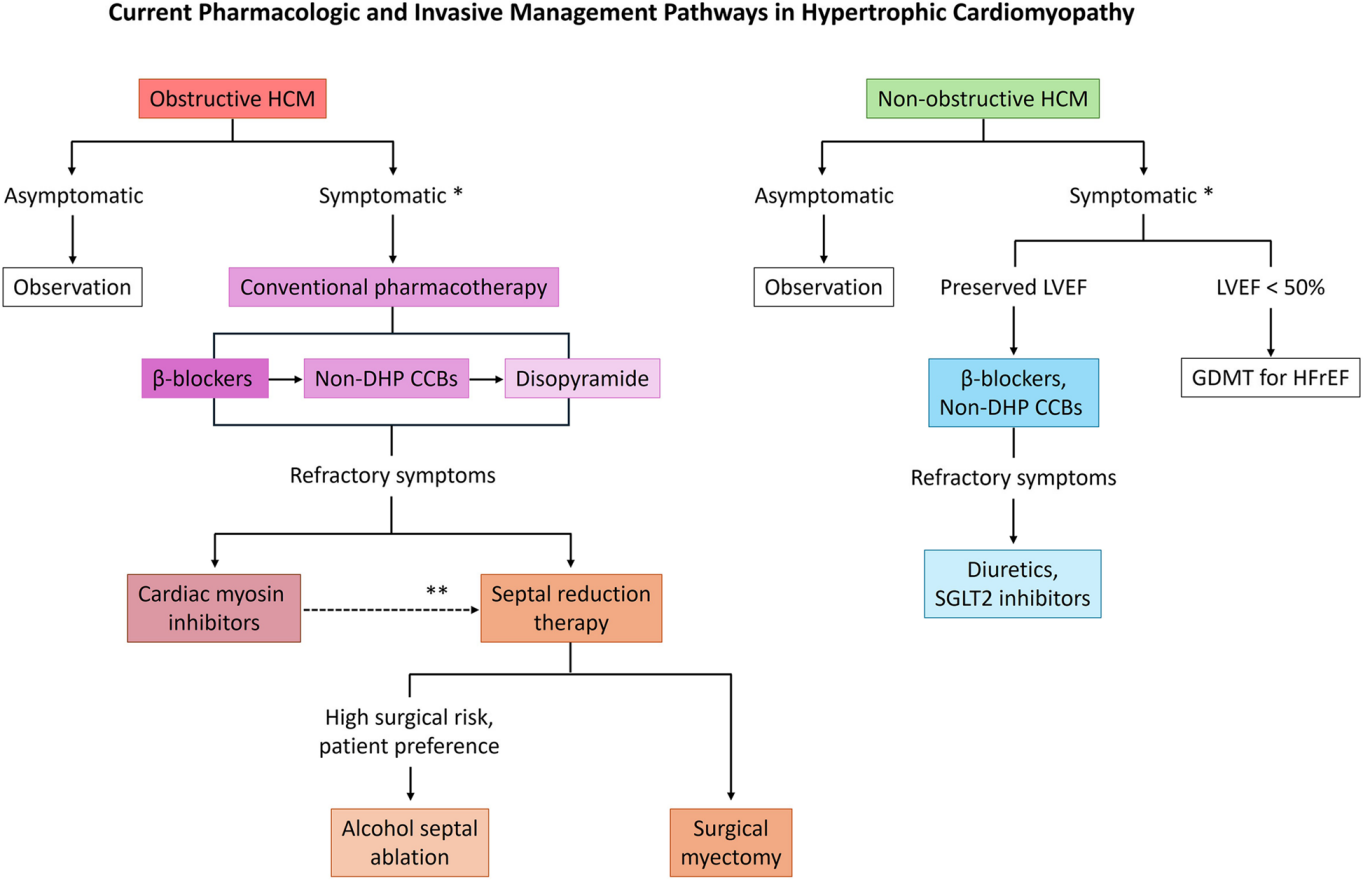
Current pharmacologic and invasive management pathways in hypertrophic cardiomyopathy (HCM). CCB, calcium channel blocker; DHP, dihydropyridine; LVEF, left ventricular ejection fraction; GDMT, guideline-directed medical therapy; HFrEF, heart failure with reduced ejection fraction; SGLT2, sodium-glucose cotransporter 2 *Symptoms due to hypertrophic cardiomyopathy include exertional dyspnea, angina, and syncope **Indicates that the comparative effectiveness of cardiac myosin inhibitors and septal reduction therapy has not been established

For many decades, SRT has been a cornerstone in the management of obstructive HCM when medications fail or are unavailable to adequately relieve symptoms. Approximately 60% to 70% of HCM patients have LVOT obstruction either at rest or with provocation, and is the responsible mechanism in the majority of patients with severe functional limitation [8, 84]. SRT is indicated in symptomatic patients (NYHA class III–IV) with significant gradients (resting or provoked peak LVOT gradient ≥ 50 mmHg) despite optimized medical therapy [4, 5]. Two main approaches are available: surgical septal myectomy (the current gold-standard) and percutaneous septal ablation, most commonly ASA, but coil or microsphere embolization of the septal artery instead of alcohol has also been reported. Endomyocardial radiofrequency ablation has also been explored as an alternative in select patients. Figure 1 summarizes the current pharmacologic and invasive therapeutic approach to the management of both obstructive and nonobstructive HCM, outlining the clinical decision-making process from initial medical therapy to advanced interventions. This section reviews these interventions, comparing their indications, techniques, outcomes, and recent data, as well as patient selection criteria and long-term considerations.

### Septal myectomy

Surgical myectomy involves direct resection of a portion of the hypertrophied interventricular septum to widen the LVOT. First performed by Andrew G. Morrow in the 1960 s, the trans-aortic septal myectomy (the Morrow procedure) has evolved into a highly effective and durable therapy for obstructive HCM [85]. The surgeon typically removes 5 to 10 g of muscle from the basal septum, often extending toward the mid-septum to the level of the papillary muscles for a more complete relief of obstruction [86–89]. In patients with midventricular obstructions, a transapical approach can be used in combination with transseptal myectomy [90–93]. Nevertheless, surgical intervention for midventricular obstruction is more limited compared to LVOT obstruction and is typically performed at highly experienced centers due to its technical complexity. Concomitant procedures are common: abnormalities of the mitral valve can be repaired including plication or resection of the elongated leaflets [89, 94–96]. If there are abnormal papillary muscle attachments, surgical mobilization or reimplantation of papillary muscles can be done [89, 96]. Of note, mitral valve replacement instead of repair is associated with worse outcomes, and should not be performed for relief of LVOT obstruction alone [5, 96]. A Cox-Maze procedure can also be performed in patients with atrial fibrillation [97]. The use of transesophageal echocardiography intraoperatively is standard to guide the location of leaflet-septal contact and extent of resection, and also to assess gradient improvement [89].

Surgical myectomy abolishes or significantly reduces the LVOT gradient in approximately 90% of patients, leading to marked symptomatic improvement [4, 98–100]. Acute hemodynamic results are typically a near-zero gradient at rest and only minimal gradient with provocation [89, 100–102]. Symptoms improve dramatically in most patients; exercise capacity and quality of life return toward normal for age. Long-term follow-up shows that most patients remain in a much-improved NYHA class years after surgery [98–103]. Survival after myectomy approaches that of an age-matched general population in some series, especially when done at high-volume HCM centers [89, 99–106]. In addition, myectomy has also shown the potential to reduce the risk of sudden cardiac death [107], and to improve diastolic dysfunction [108].

The surgical risk of septal myectomy at expert centers is low, with perioperative mortality around 0.5% in contemporary reports [89, 106, 109, 110]. Although rare, major complications include ventricular septal defects, arrhythmias, and importantly, conduction block [111–113]. Myectomy often results in a new left bundle branch block postoperatively because the resection area is near the left bundle, but does not influence postoperative mortality [114]. If a patient has preexisting right bundle branch block, there is a risk of complete heart block post-myectomy, necessitating a permanent pacemaker [101, 102]. However, overall pacemaker rates after isolated myectomy are relatively low (approximately 5% or less in most reports, unless concomitant valve surgery is done) [89, 99–102, 114, 115]. A minority of patients will experience residual or recurrent gradients due to incomplete resection, anomalies of papillary muscles and mitral valve, or midventricular obstruction, the occurrence of which may be reduced by careful preoperative planning and use of interoperative transesophageal echocardiography [89, 116, 117]. Importantly, septal myectomy is a complex procedure with a steep learning curve, and nationwide data from the United States have shown that low-volume centers have a more than a tenfold higher mortality risk compared to dedicated HCM centers, and higher complication rates including need for permanent pacemaker implantation and mitral valve replacement [115, 118–120]. Therefore, surgical myectomy should be performed in experienced HCM centers if possible [5, 118].

Lately, some centers are utilizing minimally invasive surgical approaches to reduce recovery time and complications using thoracoscopic or robotic guidance [121, 122], and even transapical beating-heart septal myectomy has been explored [123]. Cardiac computed tomography has shown the potential to improve results using three-dimensional printing to guide preoperative planning [124–126]. The threshold for intervention has also been debated—for example, intervening earlier in patients with less severe symptoms [127]. In a large retrospective study, earlier surgery (NYHA class II or impaired exercise capacity vs. the current guideline indication of NYHA class III–IV) had better long-term outcomes compared to the guideline-recommended class I indication [128]. Earlier surgery may also be considered in patients with severe pulmonary hypertension, left atrial enlargement, poor exercise capacity, or very high resting gradients [104, 129–132].

### Alcohol septal ablation

ASA is a percutaneous catheter-based technique that induces a controlled myocardial infarction in the basal septum to reduce septal thickness and alleviate LVOT obstruction. First introduced by Sigwart [133] in 1994, ASA rapidly gained acceptance as a less invasive alternative to surgery for patients with obstructive HCM. In ASA, an interventional cardiologist advances a catheter into a septal perforator artery (usually the first septal branch off the left anterior descending artery) [134]. The alcohol causes infarction and necrosis of the myocardium supplied by that branch—ideally the same area of septum that a surgeon would remove in a myectomy. Over weeks to months, the infarcted tissue scars and thins, widening the outflow tract and reducing systolic anterior motion of the mitral valve [135].

ASA is typically done under light sedation with continuous hemodynamic and echocardiographic monitoring. A temporary pacemaker is typically placed prophylactically due to the risk of atrioventricular (AV) block during the procedure [134, 136]. After engaging the targeted septal artery via coronary catheterization, the operator injects echocardiographic contrast while performing echocardiography to ensure that the contrast perfuses the correct septal region (visualized as contrast"blushing"in the basal septum on echocardiography) [134, 137, 138]. Enhancement of other regions such as the inferior wall, papillary muscles, or right ventricle is an absolute contraindication to ASA, requiring termination of the procedure [138, 139]. Once confirmed, 1 to 3 mL of 96% to 100% ethanol is slowly injected into the septal artery [136]. The alcohol causes an immediate localized infarction; the effect on gradient can often be seen acutely as septal akinesia develops [133, 135]. After injection, the artery is typically occluded using balloon inflation for a few minutes to prevent washout [137]. Patients are monitored in hospital for 3 to 5 days primarily for rhythm monitoring, as heart block can occur typically within the first 24 to 48 h [135, 138].

Appropriate patient selection is crucial for ASA success [135, 138]. Candidates are symptomatic obstructive HCM patients identical to those for myectomy, who either are suboptimal surgical candidates (older age, significant comorbidities, or patient preference to avoid surgery) [4, 5]. Coronary anatomy must be favorable: there should be a sizable septal branch that perfuses the target septal segment. If the septal perforator anatomy is not suitable (e.g., no sizable branch supplying the basal septum, or variant blood supply), ASA may not be feasible [135, 138]. ASA is less effective in patients with marked septal thickness (≥ 30 mm) or LVOT gradients ≥ 100 mm Hg [136, 140, 141]. Patients with concomitant significant valve disease or other surgical indications should be recommended surgery [4, 5].

When successful, ASA yields symptomatic improvement comparable to surgery. Gradients are reduced substantially (often by > 50%), and long-term follow-up of ASA shows that benefits are maintained in terms of symptom relief and exercise capacity [142, 143]. An important consideration is that the gradient reduction from ASA may not be immediate; while there is often an acute drop, further reductions may occur up to three months after the procedure as scar contraction develops [144]. Like myectomy, ASA appears to improve survival by relieving obstruction, and residual LVOT obstruction has been associated with worse symptom improvement and mortality [142, 145, 146]. Contemporary studies report that survival at 5 to 10 years after ASA is similar to that of post-myectomy patients and approaches that of the general HCM population, especially when done at experienced centers [136, 142, 145–147].

The most frequent complication of ASA is AV block, as the septal infarct often involves the proximal portion of the bundle branches. Transient complete heart block occurs in a significant fraction of patients; about 10% of patients will require a permanent pacemaker due to persistent AV block [134, 136, 143, 148]. Inadvertent infarction of the papillary muscles, right ventricular wall, or the left ventricular anterior wall due to collateral flow is also a potential complication [138, 149]. This may result in serious complications such as ventricular septal defect, but are infrequent when meticulous technique is used in experienced centers (early mortality approximately 1%, comparable to surgery) [142, 143]. Lower alcohol doses have been proposed to reduce complications, although at an increased possibility of repeat procedures [134, 135, 150]. There are also concerns for an increase in ventricular arrhythmia risk due to scar tissue formation from ASA [106, 151–154]; however, this issue remains controversial [138, 155, 156].

### Septal myectomy vs. ASA

The debate over whether surgical myectomy or ASA should be the primary treatment for HCM patients with refractory LVOT obstruction has been highly contentious [152, 157]. There has never been a randomized trial directly comparing septal myectomy versus ASA and likely never will be, considering the low prevalence of HCM with LVOT obstruction and the near-zero mortality rate of both procedures [158]. The majority of observational studies and meta-analyses seem to indicate comparable efficacy in symptom relief, gradient abolishment, and postoperative mortality [159–162]. However, in two large, recent studies, one from three large-volume HCM centers and another using the Medicare database, ASA was associated with higher all-cause mortality compared with myectomy, which remained significant even after adjustment for older age and a higher proportion of comorbidities in the ASA group [147, 163]. In a meta-analysis including 27 observational studies, although all-cause mortality was similar in the entire group, ASA was associated with less reduction of LVOT gradients, higher reoperation rates, and higher long-term mortality [164].

One advantage of ASA is shorter hospital stay and recovery due to a less invasive procedure, leading to natural patient preference [161]. However, experience at HCM centers of excellence have shown that when patients are fully educated about the advantages and disadvantages of each strategy, the majority will opt for surgical myectomy [152, 157]. Myectomy may achieve a greater absolute gradient reduction and is more likely to eliminate the gradient entirely; ASA’s effect can be more variable and sometimes incomplete, potentially requiring a second procedure in approximately 10% of cases [165]. Myectomy is preferred in younger patients and those with certain anatomic complexities that might not be well addressed by catheter techniques [4, 5]. Such complexities include very thick septum (approximately > 25 mm), involvement of anomalous papillary muscles or subvalvular structures, and coexistent valvular disease (e.g., intrinsic mitral valve abnormalities that can be corrected surgically during myectomy) [4, 5, 96]. Essentially, any scenario where a tailored surgical approach can address multiple abnormalities favors myectomy, as the surgeon has the advantage of direct visualization of anatomic structures which increases the possibility of an optimal result [152, 157, 166]. Surgical myectomy outcomes are known to be worse following an attempt using ASA, which should be considered in the choice of initial treatment [167].

Both the ACC/AHA and ESC guidelines give a class I indication for either SRT method in severely symptomatic obstructive HCM despite medical therapy, and acknowledge ASA as a valid alternative to surgery in appropriate patients [4, 5]. Surgical myectomy is preferred in younger patients with complex anatomy, whereas ASA is reasonable in patients who are older or with serious comorbidities or who clearly prefer a less invasive approach. A key point is that patients should be involved in a shared decision process—some may opt for surgery, while others may strongly wish to avoid open-heart surgery and accept the risk of pacemaker implantation or need for repeat intervention. Importantly, SRT should when possible be performed in high-volume/dedicated HCM centers, as postprocedural outcomes are highly dependent on center volume and the available level of expertise, although this dependence is higher for myectomy than for ASA [115, 118–120, 146]. A multidisciplinary team, similar to the Heart Team in valvular heart disease [168], should assess and discuss all patients before intervention for optimal results [4, 5, 169].

### Alternative septal reduction therapies

Alternative variations of ASA using embolization coils, foam particles, and cyanoacrylate have been reported to effectively reduce LVOT gradients [170–174]. The idea is to mechanically occlude the vessel, causing infarction by ischemia rather than chemical injury. Coiling can be an option if alcohol is contraindicated or unavailable, and it avoids any issue of alcohol leaking to unintended areas. However, embolization using these alternative methods still causes an infarct and similar precautions about AV block apply [170]. In addition, direct comparison of these methods with myectomy and ASA, as well as long-term outcome data, are not available.

Endocardial radiofrequency ablation of septal hypertrophy has also been explored as an alternative to ASA in patients with unfavorable coronary anatomy, with the rationale that induction of a localized septal contraction disorder could reduce LVOT gradients even without reduction of septal mass [175]. A number of studies have demonstrated the efficacy of radiofrequency ablation in reducing LVOT gradients [175–181]. A meta-analysis comparing septal myectomy versus radiofrequency ablation showed that although surgical myectomy was more effective in reducing septal wall thickness and LVOT gradients, improvement of symptoms was similar in both groups [182]. Predictors of a high reduction in LVOT gradients include limited basal septal hypertrophy, shorter anterior mitral leaflet, and normally positioned papillary muscle [180]. However, all publications up to date have reported results on a limited number of patients, and further study will be needed before this modality can be considered as a first-line option for obstructive HCM. Complications of radiofrequency ablation include a high proportion of complete AV block and pericardial tamponade [175]. A novel transapical, intramyocardial approach has shown safety and efficacy comparable to surgical myectomy or ASA in 200 patients, but this study was limited to a single center and remains to be validated in further trials [183].

Finally, the emergence of effective medical therapy (i.e., myosin inhibitors) must be considered in the future management of LVOT obstruction, as the VALOR-HCM trial showed that pharmacotherapy adequately relieves obstruction in a considerable proportion of patients, reducing the number of patients meeting guideline criteria for SRT [14]. Despite the growing adoption of myosin inhibitors and the longstanding use of SRT, no studies to date have directly compared these approaches in patients with obstructive HCM. Given their fundamentally different mechanisms—pharmacologic modulation of contractility versus anatomical relief of obstruction—rigorous head-to-head trials are needed to guide individualized treatment strategies. However, conducting such trials may be challenging in practice. As an alternative, comparative effectiveness research using real-world data or large-scale epidemiologic cohorts may provide supportive insights into the relative benefits and limitations of each approach. Future research should prioritize comparative effectiveness studies that incorporate clinical outcomes, myocardial remodeling, and long-term durability of benefit. Until more definitive evidence becomes available, treatment decision should be individualized based on the patient’s anatomical features, comorbidities, and demographic background. A multidisciplinary approach is essential to determine the most appropriate therapy. For instance, in younger patients or those with limited financial resources—who may face challenges related to the high long-term cost of myosin inhibitors despite reimbursement—surgical myectomy may serve as a more definitive and cost-effective option. Conversely, for patients who refuse invasive procedures or are at high procedural risk, myosin inhibitors may be considered as a first-line treatment. Ultimately, shared decision-making within a multidisciplinary framework is critical to align therapeutic strategies with patient preferences and individualized clinical and anatomical considerations.

## Conclusions

**Fig. 2:**
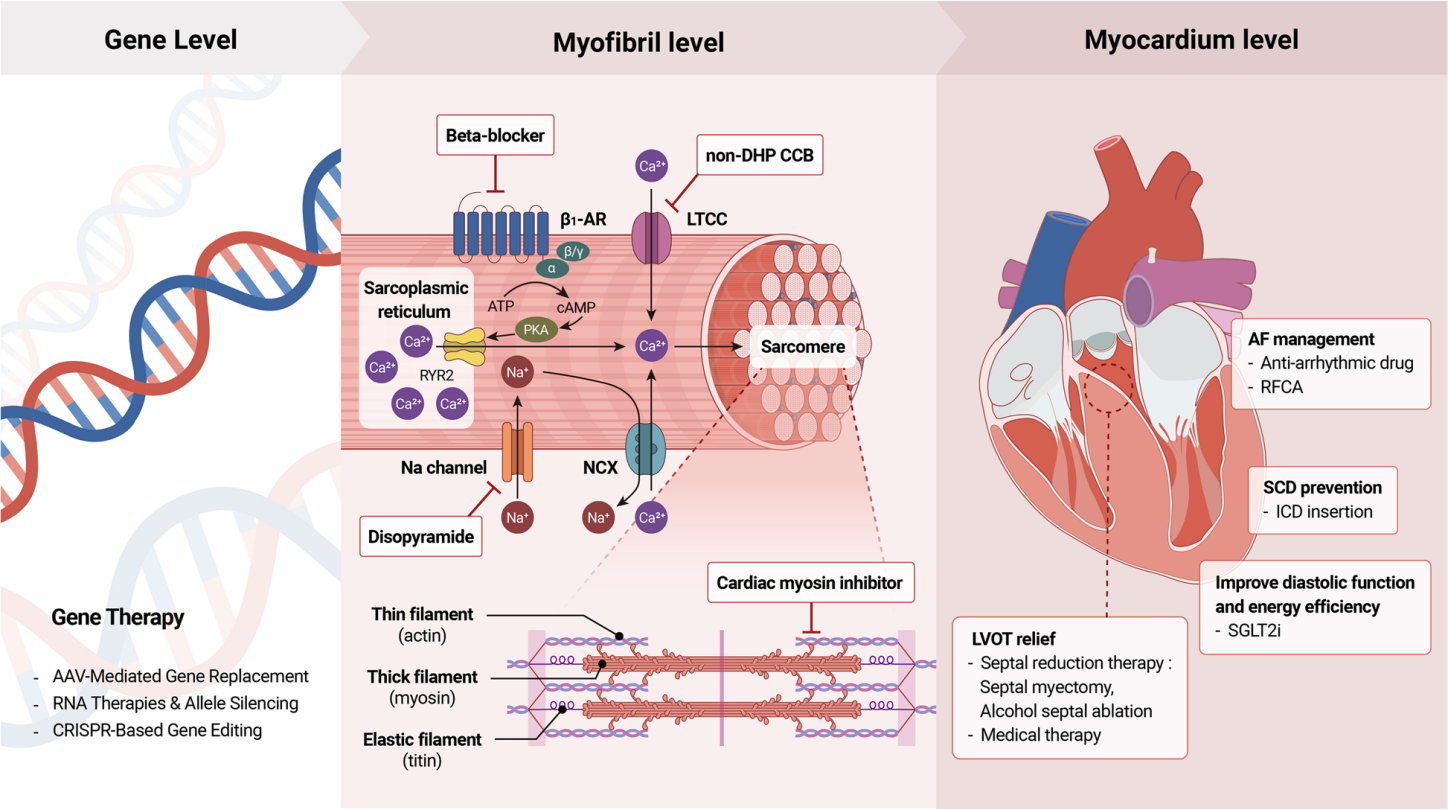
Multilevel treatment strategies in hypertrophic cardiomyopathy (HCM). Current management strategies in HCM target different levels of disease pathophysiology. At the gene level, investigational approaches such as adeno-associated virus (AAV)-mediated gene replacement, RNA-based therapies, and CRISPR (clustered regularly interspaced short palindromic repeats)-based gene editing are under development to correct or silence pathogenic variants. At the myofibril level, pharmacologic therapies—including β-blockers, nondihydropyridine (non-DHP) calcium channel blockers (CCBs), disopyramide, and cardiac myosin inhibitors—aim to improve symptoms by modulating sarcomeric function and calcium handling. At the myocardium level, four major therapeutic targets are addressed: (1) relief of left ventricular outflow tract obstruction (LVOTO) through septal reduction therapy—including surgical myectomy and alcohol septal ablation—and pharmacologic therapies that act at the myofibril level (as described above); (2) improvement of diastolic function and myocardial energy efficiency with agents such as sodium-glucose cotransporter 2 inhibitors (SGLT2i); (3) management of atrial fibrillation (AF) using antiarrhythmic drugs or radiofrequency catheter ablation (RFCA); and (4) prevention of sudden cardiac death (SCD) with implantable cardiac defibrillator (ICD) insertion. This layered approach illustrates the evolving paradigm of integrating established treatments with emerging targeted therapies in HCM. ATP, adenosine triphosphate; β1-AR, β1 adrenergic receptor; cAMP, cyclic adenosine monophosphate; LTCC, L-type calcium channel; NCX, sodium-calcium exchanger; PKA, protein kinase A; RYR2, ryanodine receptor 2

The management of HCM is evolving from a focus on symptom relief and sudden cardiac death prevention toward a more mechanism-targeted approach spanning from gene to myocardium (Fig. 2). Established therapies, including pharmacologic agents, SRT, and implantable cardiac defibrillator insertion, remain central to care. Recent advances such as SGLT2 inhibitors, cardiac myosin inhibitors, and investigational gene-targeted therapies offer promising new strategies, although these require further validation. Ongoing research will be essential to define how these emerging treatments can be effectively integrated into personalized, multidisciplinary management of HCM.

## Data Availability

No datasets were generated or analysed during the current study.

## Abbreviations

AAV: Adeno-associated virus
ABE: Adenine base editor
ACC: American College of Cardiology
AHA: American Heart Association
ASA: Alcohol septal ablation
ASO: Antisense oligonucleotide
ATP: Adenosine triphosphate
AV: Atrioventricular
CCB: Calcium channel blocker
Cas9: CRISPER-associated protein 9
CRISPR: Clustered regularly interspaced short palindromic repeats
ESC: European Society of Cardiology
HCM: Hypertrophic cardiomyopathy
LVOT: Left ventricular outflow tract
NYHA: New York Heart Association
SGLT2: Sodium-glucose cotransporter-2
SRT: Septal reduction therapy
